# Cardiac changes associated with vascular aging

**DOI:** 10.1002/clc.23313

**Published:** 2019-12-16

**Authors:** Narayana Sarma V. Singam, Christopher Fine, Jerome L. Fleg

**Affiliations:** ^1^ Division of Cardiovascular Medicine University of Louisville Louisville Kentucky; ^2^ Division of Cardiovascular Sciences National Heart, Lung, and Blood Institute Bethesda Maryland

**Keywords:** aging and the cardiovascular system, cardiac mechanics, echocardiography, heart failure, remodeling

## Abstract

Cardiovascular aging is a complex process of adaptive structural and functional changes over time. With advancing age, the arterial tree thickens and decreases in compliance, resulting in increased pulse wave velocity, systolic blood pressure, and left ventricular afterload. In response to these arterial changes, the myocardium remodels to maintain systolic function and diastolic filling. These adaptive mechanisms are not necessarily pathologic but increase the susceptibility for myocardial ischemia and heart failure in the presence of common age‐associated comorbidities. This article reviews the pathophysiology of cardiovascular aging and discusses therapeutic interventions that may ameliorate these processes.

## 1 BACKGROUND

By the year 2060, the number of individuals 65 years and older in the United States is expected to double to 96 million and will constitute nearly 23% of the population.^1^ Although cardiovascular (CV) disease is not necessarily precipitated by advanced age, it is the most significant contributor to morbidity and mortality in older adults.^2^ Moreover, the elderly are at elevated risk for developing heart disease due to an increased prevalence of age‐associated comorbidities such as hypertension, diabetes mellitus, and dyslipidemia. Thus, the incidence of coronary artery disease (CAD), valvular disease, rhythm disorders, and heart failure increases with age.^2^ There is keen interest, therefore, in understanding the changes in CV physiology that occur with advancing age and how they may contribute to the development of clinical CV disease. Many of these changes begin in early adulthood, but they typically become clinically relevant at older ages. This review will explore the effects of age on CV pathophysiology, how they predispose to disease, and therapeutic interventions that may attenuate these maladaptive processes.

## 2 VASCULAR COMPLIANCE/ARTERIAL STIFFNESS

Several important changes in arterial structure and function occur with advancing age. This section will describe the effects of aging on each of these processes.

**Figure 1:**
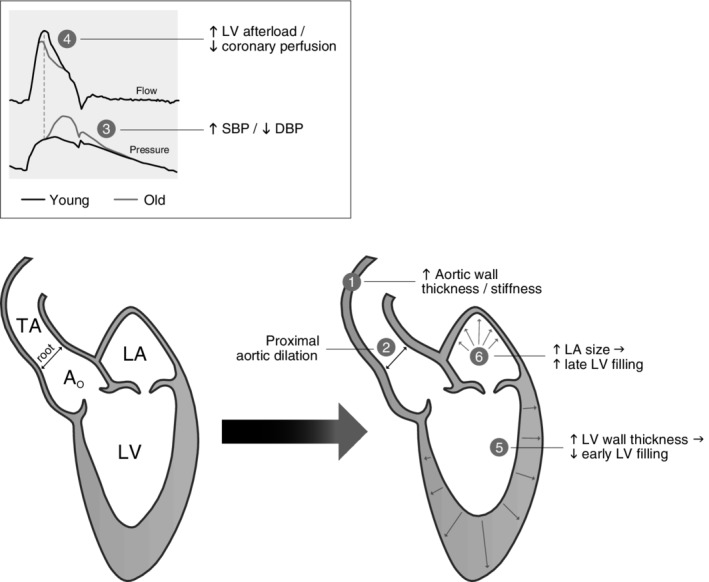
Conceptual framework of age‐related changes in cardiovascular structure and function. Abbreviations: DBP, diastolic blood pressure; EDV, end‐diastolic volume; LA, left atrium; LV, left ventricle; LVEF, left ventricular ejection fraction; PP, pulse pressure; SBP, systolic blood pressure

Vascular compliance is the ability of a blood vessel to change in cross‐sectional area (eg, distend) in response to dynamic intramural pressures. Vascular compliance can be calculated by the equation Δ*C* = Δ*V*/Δ*P*, where *C* equals compliance, *V* equals volume, and *P* equals the pressure. Compliance is directly proportional to volume and inversely related to changes in pressure. Vascular elastance, which is the “recoil tendency” of a vessel, is the reciprocal of compliance; therefore, a decrease in compliance results in an increase in elastance. With aging, arterial walls thicken due to reductions in elastin and increases in nondistensible collagen deposition.^3^ Subsequently, this stiffened artery is less able to distend in the presence of increased pressures. With each myocardial contraction, a pressure wave (ie, pulse wave) generated by pulsatile blood flow is propagated up the ascending aorta and down the large arteries, which is then reflected centrally in diastole. Pulse wave velocity (PWV) describes the velocity component of this pressure wave. In the presence of increased arterial stiffness, the pulse wave is reflected downstream more rapidly and returns before initiation of diastole, leading to augmentation of late systolic blood pressure (Figure 1).^4^ This late peak in systolic blood pressure occurs well after peak arterial flow in older adults whereas the pressure and flow peaks occur simultaneously in the young, resulting in greater CV efficiency. Novel research by Rogers et al indicates that there are also regional variations of PWV, with the proximal aorta affected more with age.^5^ Although PWV generally increases with age even in healthy populations, individuals with fewer CV risk factors will have lower PWV at a given age than those with a higher risk factor profile.^6^, ^7^ Higher PWV in older adults is also associated with cognitive impairment.^8^ The clinical manifestation of this age‐associated increase in arterial stiffness is hypertension, which most commonly presents as isolated or predominant elevation of systolic blood pressure and accelerates the development of atherosclerosis.^9^ Thus, elevated systolic blood pressure in older adults increases the risk of myocardial infarction, stroke, heart failure, and renal dysfunction. When hypertension is coupled with other risk factors such as concurrent dyslipidemia, obesity, and diabetes, it results in a more rapid progression of atherosclerosis and increased mortality. Similarly, the metabolic syndrome accelerates age‐related reduction in vascular compliance and increased arterial stiffness.^10^ In addition, the stiffened aorta is less able to distend in early diastole, resulting in a decrease of diastolic blood pressure and widening of the pulse pressure.^11^ Increased pulse pressure is an independent predictor for CV mortality, likely because coronary filling occurs during diastole and decreased diastolic pressure leads to lower coronary perfusion pressure.^11^

Ventricular afterload is another component of arterial mechanics that increases with age. Although a complex parameter, ventricular afterload can be described as the summed stress the myocardial fibers must overcome to complete ejection. In vitro models describe load as the weight (ie, force) that papillary muscles can lift. Therefore, afterload can be approximated by the pressure (ie, force per unit area) applied by the left ventricle (LV) muscle fibers to eject blood into the arterial tree. Ventricular afterload can be estimated by a simplified form of the Young‐Laplace equation, *T* = *P* × *r*/*h*, where *T* equals wall tension (impedance), *P* equals LV systolic pressure, *r* equals LV radius, and *h* equals LV thickness. Arterial impedance, the pulsatility component of arterial afterload, is the ratio of pressure to flow at a given harmonic frequency.^12^ Myocardial fibers need to overcome this arterial impedance to eject blood, and therefore, impedance is a reasonable estimate of afterload. Changes in aortic impedance directly affect the ability of the LV to unload in systole.^13^ With aging, there is an increase in degradation of the extracellular matrix (ECM) of the vascular wall, increased collagen and calcium deposition, and reduction of elastic lamellae, leading to intimal thickening and vascular dilatation.^3^ The consequence is a decrease in arterial compliance, an increase in impedance, and an increase in ventricular afterload. Although intimal thickening may occur in individuals without atherosclerotic CV disease, increased arterial wall thickness remains an independent predictor for the development of atherosclerosis.^13^ Prior studies have shown that there is active LV remodeling in the presence of increased aortic impedance with decreased LV compliance and increased LV elastance.^14^, ^15^

## 3 CARDIAC STRUCTURE AND FUNCTION

Presumably in response primarily to the arterial aging changes described above, the myocardium remodels over time. The overall number of cardiac myocytes decreases with age due to apoptosis, possibly driven by an underlying chronic inflammatory state. However, the myocyte size increases, likely in compensation for the reduced myocyte number, leading to LV hypertrophy.^16^ Studies investigating aging changes in LV size, geometry, and wall thickness have demonstrated an increased wall thickness, preserved short axis length, but decreased LV long axis length, with the resultant effect of preserved LV mass with age in women and a modest decrease in men.^17^, ^18^ Thus, advancing age is accompanied by a change in LV geometry from a conical to a more spherical shape.^3^, ^18^

Left ventricular ejection fraction (LVEF), the most commonly used measure of systolic function, is preserved across the adult age span in persons without clinical heart disease.^16^ Novel methods of strain, i.e., deformation, analysis now enable detection of subclinical LV dysfunction before a reduction occurs in LVEF. Strain analysis provides the ability to differentiate active from passive myocardial segment movement, assess ventricular dyssynchrony, and evaluate global/segmental myocardial function. There is a decline in transmural global longitudinal strain but an increase in global circumferential strain with age.^19^, ^20^ The increase in circumferential strain is likely a compensatory mechanism to maintain global LVEF.

In contrast to the overall preservation of systolic LV function with aging, diastolic LV function undergoes pronounced alterations. With advancing age, there is a decrease in peak early diastolic filling (echocardiographic E‐wave) and increased reliance on left atrial contraction (A‐wave) to maintain LV filling.^21^ This accentuated A‐wave is accomplished via a modest increase in left atrial volume.^3^ However, increased left atrial size contributes toward a lower threshold for developing atrial fibrillation and subsequent loss of A‐wave. With advancing age, therefore, individuals with atrial fibrillation are prone to decompensation during stress because this late atrial contribution to LV filling is lost, and diastolic filling is compromised. As a result, older adults who develop atrial fibrillation are more susceptible to the development of heart failure, typically with preserved ejection fraction (HFpEF).^22^ Figure 1 provides a conceptual framework for the major CV structural and functional changes that occur at rest with advancing age.

Alterations in cardiac electrophysiology with age are largely attributed to calcification of the cardiac skeleton resulting in electrical conduction delay.^23^ This may manifest on the resting electrocardiogram (ECG) as atrioventricular or intraventricular delay or block. A similar process of calcification may occur in the aortic valve in older adults, resulting in calcific aortic stenosis. Increased P‐R interval, leftward shift of the QRS axis, and decreased voltage of both QRS complexes and T waves are commonly observed on the ECG with advancing age and show no consistent association with mortality or CV events.^23^ Additionally, there is an increased incidence of atrial and ventricular arrhythmias with aging, seen at rest,^24^ during ambulatory monitoring,^25^ and with exercise.^23^ The prognostic significance of these arrhythmias in apparently healthy older adults is controversial and is likely dependent on the presence or absence of subclinical CV disease.^23^

## 4 CARDIAC RESPONSE TO STRESS

The aging myocardium responds to external stressors by using a variety of compensatory mechanisms. Hemodynamic parameters such as blood pressure and heart rate are blunted or accentuated, depending on the specific stress employed. The hemodynamic adjustments made with stress are often useful in the assessment of CV health and diagnosis of subclinical disease.

### 4.1 CV response to posture

In healthy older adults, there is a blunted heart rate response to assumption of upright posture. The reduced heart rate response is likely due to an age‐related reduction in baroreceptor sensitivity and decline in cardiac beta‐receptor density, given the documented increase in circulating levels of catecholamines, coupled with a reduction in neurotransmitter reuptake, during orthostatic stress or exercise.^13^, ^26^, ^27^ Another observed physiologic change with age is a sluggish carotid baroreceptor reflex induced by a reduction in pressures distal to the aortic arch.^28^ These processes likely play a role in the increased incidence of orthostatic hypotension (OH), defined by a decline in systolic blood pressure by ≥20 mm Hg or diastolic blood pressure ≥ 10 mm Hg, in advanced age. The Cardiovascular Health Study and Honolulu Heart Program have demonstrated OH to occur in 16% of community volunteers older than 65 years and 17% of men aged 71‐93 years, respectively.^29^, ^30^ In some studies, OH is an independent predictor of increased mortality, with the magnitude of orthostatic BP decline linearly correlated with 4‐year mortality.^29^, ^30^ Supine hypertension, increased LV wall thickness, and a small LV cavity size are predictive factors for OH in older adults.^31^

### 4.2 CV response to afterload stress

With advanced age, the contractile reserve of the LV decreases with acute increases in systemic blood pressures elicited during sustained handgrip maneuvers as well as low‐dose phenylephrine infusion under concurrent beta‐blockade.^32^, ^33^ Under these conditions, echocardiography demonstrated increased systolic and diastolic LV dimensions, and an increased reliance on late diastolic filling in older adults.^32^, ^33^ Moreover, acute changes in systemic afterload highlight age‐associated dependence on late diastolic filling to preserve stroke volume and maintain cardiac output.

### 4.3 CV response to exercise

The LV of a healthy older adult maintains a normal resting LVEF but has blunted augmentation of LVEF with aerobic exercise.^16^ There are many proposed mechanisms for this phenomenon. Contributing factors may include reduced intrinsic myocardial contractility, blunted arterial vasodilator capacity leading to increased cardiac afterload, and reduced beta‐adrenergic responsiveness with aging. Augmentation of LVEF during exercise is further impaired in individuals with exercise‐induced silent myocardial ischemia.^34^ Although each of these factors by itself is unlikely to explain the development of clinical heart failure, in concert, they may lower the threshold for symptom development.^35^

Variations in load‐bearing capabilities of the LV and the systemic arterial vasculature contribute to exercise‐induced CV dysfunction in older adults.^26^ For the LVEF to augment during exercise, the LV end‐systolic elastance (ie, the ratio of end‐systolic pressure to end‐systolic volume) must increase beyond that of the analogous elastance of the vascular bed. If it does not, the LVEF response to exercise is blunted, resulting in lower cardiac output and an increase in LV end‐systolic diameter. These individuals may be more susceptible to developing clinical heart failure. Ventricular‐vascular coupling, the relationship between the LV and the distal vasculature, leads to optimal ejection of blood when loading conditions are matched. This relationship remains preserved in normal aging at rest given the proportional increase in arterial and ventricular stiffness. However, with exercise in older individuals, the LV end‐systolic elastance fails to increase in parallel with the arterial elastance. This suboptimal load matching ratio during exercise results in a decreased LVEF reserve compared to that in younger adults.^23^, ^35^

The sympathetic nervous system also plays an integral role in effective CV modulation during exercise. It regulates increases in heart rate, changes in myocardial inotropy and relaxation, systemic vasodilation, and redistribution of arterial blood flow to necessary organs. When considering multiple scientific perspectives (eg, intact human models and biochemical cascades in animal models), a decreased response of beta‐adrenergic modulation has proven to be the most consistently observed CV physiologic response that occurs with advancing age.^36^ The boundary between healthy and pathologic aging is crossed when age‐associated “normative changes” occur in conjunction with severe deconditioning and CV disease, both of which are highly prevalent in older adults.

## 5 MOLECULAR AND CELLULAR CHANGES

### 5.1 Endothelial dysfunction

It is well established that oxidative stress is increased with advancing age through the release of reactive oxygenation species produced by nicotinamide adenine dinucleotide phosphate (NADPH) oxidase leading to mitochondrial dysfunction and a maladaptive endothelial function.^28^ Chronic inflammatory markers such as tumor necrosis factor‐α and interleukin‐6 increase within vascular and myocardial tissue over time and are thought to be a consequence of redox‐sensitive cellular signaling pathways.^37^ These factors down‐regulate the production of nitric oxide synthase and subsequently reduce the production of nitric oxide (NO) in older adults. Low levels of NO inhibit physiologic vasodilatation and cause a chronic inflammatory state within the CV system leading to a decrease in myocardial compliance. Furthermore, low NO decreases protein kinase G activity within myocytes, leading to LV hypertrophy and increased stiffness.^38^

### 5.2 Extracellular matrix

In addition to myocyte hypertrophy, the cardiac ECM also thickens with age due to increased deposition of collagen mediated by profibrotic agents.^3^ With renal aging, there is an elevated activation of the vascular renin‐angiotensin‐aldosterone‐system (RAAS), leading to increased levels of angiotensin II in the arterial wall. The downstream effect of RAAS activation is the upregulation of tumor growth factor‐beta (TGF‐β), which subsequently increases collagen production and deposition into ECM, resulting in vascular thickening.^39^ Furthermore, oxidative stress also upregulates matrix metalloproteinase development and further increases vascular fibrosis leading to LV remodeling and subsequent deleterious CV aging.^40^, ^41^ Table 1 summarizes the common age‐associated changes that occur in the CV system and their potential relationship to developing CV disease.^16^

**Table 1:** Relationship of cardiovascular aging to disease

Age‐associated changes	Plausible mechanisms	Possible relationship to disease
CV structural remodeling		
↑ Vascular Intimal Thickness	↑ VSMC migration and matrix production	Early stages of atherosclerosis
↑ Vascular stiffness	Elastin fragmentation ↑ Elastase activity ↑ Collagen production and cross‐linking	Systolic hypertension
	Altered growth factor regulation,and tissue repair	Atherosclerosis
↑ LV wall thickness	↑ LV myocyte size ↓ Myocyte number ↑ Focal collage deposition	↓ Early LV diastolic filling ↑ LV filling pressure and subsequent dyspnea
↑ Left atrial size	↑ Left atrial volume/pressure	↑ Risk of atrial fibrillation
**CV functional changes**		
Altered vascular tone	↓ NO production/effects ↓ βAR responses	Vascular stiffening and systolic hypertension
↓ CV reserve	↑ Vascular load ↓ Intrinsic myocardial contractility ↓ β‐adrenergic modulation of heart rate, LV contractility and vascular tone	Increased susceptibility for heart failure
↓ Physical activity	Comorbidities	Accelerated aging changes in CV structure and function
	↓ Skeletal muscle mass	↑ Risk of CV disease

Abbreviations: βAR, beta‐adrenergic receptor; CV, cardiovascular; LV, left ventricular; NO, nitric oxide; VSMC, vascular smooth muscle cell. From Reference 16.

*Source*: Adapted from Reference 16.

### 5.3 Amyloid infiltration

In addition to the myocardial structural changes mentioned above, there is also an increased incidence of misfolded protein deposition in the aged heart. Mutated wild‐type transthyretin protein has been detected in up to 25% of heart autopsies in older adults.^42^ With large amounts of myocardial infiltration, this mutated protein leads to the wild‐type transthyretin cardiac amyloidosis (ATTRwt), previously known as senile cardiac amyloidosis. With advances in echocardiography, cardiac MRI, and nuclear imaging, ATTRwt is becoming more widely recognized in older adults.^43^ This infiltrative cardiomyopathy can cause HFpEF with LV hypertrophy that can be identified on echocardiography in association with low‐QRS voltage on ECG. Such patients often exhibit sinoatrial block, atrioventricular block, bundle branch block, and atrial arrhythmias. Cardiac MRI and technetium scintigraphy can aid in the diagnosis. Although the prognosis for ATTRwt has traditionally been poor, novel therapeutic agents such as tafamidis, which stabilizes transthyretin, preventing its dissociation into amyloid, and patisiran, which interferes with amyloid production, can improve prognosis.^44^, ^45^

## 6 POTENTIAL INTERVENTIONS FOR CV AGING

Over the past half‐century, significant advances have been made in understanding the physiology of aging. Although advances in therapeutics to reduce the rate of aging in humans have been limited, interventions such as caloric restriction, exercise training, and pharmacological agents show promise in blunting aging changes in the CV system and elsewhere.

It has long been established in animal models that caloric restriction extends longevity and may attenuate CV aging changes.^46^ Data from the Comprehensive Assessment of Long‐Term Effects of Reducing Intake of Energy (CALERIE) study indicates that 2 years of moderate caloric restriction in healthy, nonobese adults 21‐50 years old reduces the rate of biologic aging, including lowering blood pressure, increasing insulin sensitivity, and improving lipid profiles.^47^, ^48^ Whether caloric restriction is similarly effective at older ages remains unproven but worthy of future evaluation. At a cellular level, caloric restriction increases phosphorylation and activation of AMP‐activated protein kinase (AMPK) which in turn triggers autophagia, an intrinsic process of cellular regeneration, which is known to be cardioprotective against myonecrosis via ischemic preconditioning.^49^, ^50^

It is known that oxidative stress promotes pathologic aging by decreasing NO production in endothelial cells, leading to impaired vasodilation, atherogenesis, and increases in apoptosis. The resultant endothelial dysfunction contributes to hypertension and increased atherosclerosis among other pathologic changes.^51^ Pharmacologic strategies with antioxidants are being explored to reduce these adverse effects. Resveratrol (3,4′,5‐trihydroxy‐trans‐stilbene), a polyphenol commonly found in wine and grapes, has been implicated in disrupting the oxidative pathways of aging.^52^, ^53^ Although the exact mechanism of action is unclear, resveratrol appears to activate AMPK (similar to caloric restriction) and sirtuin‐1 (SIRT1) production. Sirtuin‐1 is an enzyme that protects against apoptosis and in moderately overexpressed concentrations appears to play a cardioprotective role in aging.^54^ Furthermore, a Mediterranean diet (emphasizing whole grains, vegetables, fruits, olive oil, beans, and nuts) provides antioxidative and anti‐inflammatory effects in aging animals by increasing NO bioavailability in endothelial cells, leading to decreased apoptosis.^55^ Research is ongoing on the effects of antioxidant vitamins E and C on CV aging. The landscape of dietary supplements and foods will likely evolve over the next decade as further research elucidates the antioxidant and anti‐inflammatory processes that blunt adverse aging mechanisms.

Exercise is another potent attenuator of age‐related CV changes. Regular moderate‐intensity aerobic exercise has been shown to reduce body fat, weight, and blood pressure, improve insulin sensitivity, increase endothelial function, and improve maximal oxygen uptake.^56^ A recent randomized prospective study by Howden et al, noted a reduction in LV myocardial stiffness in middle‐aged adults after 2 years of rigorous supervised aerobic exercise training.^57^ Conversely, a sedentary lifestyle has been associated with increased LV stiffness, impaired insulin sensitivity, and a higher incidence of HFpEF.^57^, ^58^

## 7 CONCLUSIONS

The CV system undergoes structural and functional changes over the adult lifespan. With advancing age, the arterial tree becomes stiffer, leading to higher systolic blood pressure and pulse pressure, and a higher risk of systolic hypertension, which predisposes to LV hypertrophy to maintain a normal LVEF. As a compensatory mechanism for reduced early diastolic filling rate, the older heart becomes more reliant on late diastolic filling from the left atrium. These maladaptive processes may predispose an older individual to myocardial ischemia and heart failure. Deposition of mutated proteins over time increases the prevalence of ATTRwt in the elderly. Blunted CV responses to postural, exercise, and afterload stress are also characteristic of advancing age. Recent studies of caloric restriction, exercise training, and antioxidants have shown promise to decrease the rate of CV aging. Further research in this field will have important implications for reducing the incidence of clinical CV disease that so commonly occurs in older adults.

## DISCLAIMER

The views expressed in this review are those of the authors and do not necessarily represent those of the National Heart, Lung, and Blood Institute, the National Institutes of Health, or the United States Department of Health and Human Services.

## CONFLICT OF INTEREST

The authors declare no potential conflict of interests.
